# The Controversial Role of PD-1 and Its Ligands in Gynecological Malignancies

**DOI:** 10.3389/fonc.2019.01073

**Published:** 2019-10-15

**Authors:** Oliviero Marinelli, Daniela Annibali, Cristina Aguzzi, Sandra Tuyaerts, Frédéric Amant, Maria Beatrice Morelli, Giorgio Santoni, Consuelo Amantini, Federica Maggi, Massimo Nabissi

**Affiliations:** ^1^School of Pharmacy, University of Camerino, Camerino, Italy; ^2^School of Bioscience and Veterinary Medicine, University of Camerino, Camerino, Italy; ^3^Gynecological Oncology, Oncology Department, LKI Leuven Cancer Institute KU Leuven-University of Leuven, Leuven, Belgium; ^4^Centre for Gynecologic Oncology Amsterdam (CGOA), Antoni Van Leeuwenhoek-Netherlands Cancer Institute (AvL-NKI), University Medical Center (UMC), Amsterdam, Netherlands; ^5^Department of Molecular Medicine, Sapienza University, Rome, Italy

**Keywords:** PD-L2, PD-L1, PD-1, ovarian cancer, endometrial cancer, cervical cancer, immunotherapy

## Abstract

The programmed death-1 (PD-1, CD279) receptor with its ligands, programmed death ligand 1 (PD-L1, CD274, B7-H1), and programmed death ligand 2 (PD-L2, CD273, B7-DC), are the key players of one of the immune checkpoint pathways inhibiting T-cell activation. PD-L1 and PD-L2 are expressed in different cancer cells and their microenvironment, including infiltrating immune cells. However, their prognostic value is still debated and their role in the tumor microenvironment has not been fully elucidated yet. Considering the importance that cancer immunotherapy with anti-PD-1 and anti-PD-L1 antibodies gained in several tumor types, in this review article we aim to discuss the role of the PD-1/PD-L1/PD-L2 axis in gynecological cancers. PD-1 ligands have been detected in ovarian, cervical, vulvar and uterine cancers, and correlation with prognosis seems dependent from their distribution. About PD-L2, very few reports are available so far in gynecological malignancies, and its role is still not completely understood. Clinical trials using anti-PD-1 or anti-PD-L1 antibodies, but not anti-PD-L2, are currently ongoing, in all types of gynecological cancers. They have shown good safety profiles in a certain cohort of patients, but response rates remain low and many aspects remain controversial. In this review, we propose possible solutions to enhance the clinical efficacy of PD-1 axis targeting therapies. Regarding PD-L2, it might be useful to better clarify its role in order to improve the efficiency of immunotherapy in female malignancies.

## Introduction

### PD-1 and Its Ligands, PD-L1 (B7-H1) and PD-L2 (B7-DC)

Programmed death-1 (PD-1, CD279) receptor and its ligands, programmed death ligand 1 (PD-L1, CD274, B7-H1) and programmed death ligand 2 (PD-L2, CD273, B7- DC), play crucial roles in one of the immune checkpoint pathways responsible for the inhibition of T-cell activation (1).

PD-1 receptor belongs to the CD28 family and is mainly expressed on the cellular surface of activated T and B cells, monocytes, natural killer (NK), and dendritic cells (DCs), with a role in the induction and maintenance of peripheral tolerance and for the maintenance of the stability and the integrity of T cells (2–5). PD-1 ligands are glycoproteins, members of the B7 family, with 40% homology in amino acids sequence, but have quite distinct expression patterns, being expressed by a wide variety of immune and non-immune cells (1, 3, 4).

PD-L1 is a type I transmembrane glycoprotein with a single N-terminal immunoglobulin variable (IgV)-like domain sharing 21–33% sequence identity with CTLA-4, CD28, and ICOS, about 20 amino acids that separate the IgV domain from the plasma membrane, a transmembrane domain and a cytoplasmic tail (4). It is constitutively expressed on activated T and B cells, DCs, macrophages, mesenchymal stem cells, and bone marrow-derived mast cells (4, 6). Additionally, it is expressed on a wide variety of non-hematopoietic cells including the vascular endothelium, fibroblastic reticular cells, keratinocytes, lung, non-parenchymal cells of the liver, mesenchymal stem cells, pancreatic islet cells, astrocytes, and neurons (4, 5, 7). PD-L1 expression on human T cells is induced by common γ chain cytokines (IL-2, IL-7, and IL-15), whereas PD-L1 expression on B cells is stimulated by IL-21 (4). In cancer cells, PD-L1 expression is regulated by the MAPK and PI3K/AKT pathways, as well as by HIF-1α, STAT-3, NF-κB and epigenetic mechanisms via microRNAs (8). PD-L1 also exists in a soluble form (sPD-L1) that originates from the cleavage of membrane-bound PD-L1 by matrix metalloproteinases. Such PD-L1 soluble isoform, mainly produced by myeloid-derived cells, retains the IgV-like domain, necessary for the interaction with PD-1, and it is able to suppress T-cell activation. However, its physiological role is still unknown. Interestingly, sPD-L1 has been found in several human cancer cell lines, including H1299 non-small cell lung cancer cells, U-937 lymphoma cells, HO8910 ovarian carcinoma cells, SPCA-1 lung adenocarcinoma cells and U251 glioblastoma cells. In addition, high plasma levels of sPD-L1 have been associated with metastasis and poor prognosis in breast cancer and diffuse large B-cell lymphoma (8).

PD-L2 is a type I transmembrane protein containing an IgV-like domain and an immunoglobulin constant (IgC)-like domain in its extracellular region (9). PD-L2 expression is mainly restricted to antigen-presenting cells (APCs), including macrophages and myeloid DCs (6, 7), and non-hematopoietic tissues, such as the lung (10), human umbilical vein endothelial cells, and fibroblasts (1, 5). Three isoforms of PD-L2 have been described that might influence the outcome of the immune response (9). The most common splice variant contains all 6 exons. In humans, an alternative variant with a spliced-out exon 3, resulting in a protein lacks the IgC-like domain and with a shorter—extracellular region has been reported. A third isoform misses the transmembrane domain, because exon 3 is spliced out to an alternative acceptor site within exon 4, and the protein is secreted as a soluble form. This evidence underscores the importance of post-transcriptional regulation in the expression and function of PD-L2. He et al. suggested that isoforms II and III should be able to interact with PD-1, but further confirmation is needed (9).

Exposure to IL-4, IFN-γ, IL-2, IL-7, IL-15, IL-21, and toll-like receptor ligands induces PD-L2 upregulation in DCs and macrophages (1). Additionally, IL-4, in the presence of respiratory syncytial virus infection, stimulates PD-L2 expression in alveolar epithelial cells (1, 10).

Stimulation by tumor necrosis factor alpha (TNF-α) and interferon gamma (IFN-γ) enhances the constitutive expression of PD-L2 on endothelial cells from human umbilical vein *in vitro* (1). The NF-κB and the STAT-6 pathways are two major signaling reported to regulate PD-L2 expression (1).

Different molecular mechanisms dictate PD-Ls binding to PD-1, as demonstrated by the crystallographic structures of the complexes, showing that PD-Ls cross-compete and that the concurrent presence of both ligands might modify the functional outcome of the binding (11). Specifically, PD-L1 binding to PD-1 requires complex conformational changes of the ligand, while PD-L2 directly interacts with PD-1, explaining its reported 2 to 6-fold higher affinity for the receptor (1). Consequently, when both ligands are expressed at similar levels, PD-L2 would be expected to outcompete PD-L1 for binding to PD-1. However, PD-L2 is generally expressed at lower levels in physiological conditions, such as during maturation of DCs by LPS, when PD-L1 acts as the main ligand of PD-1. A known exception is Th2 responses, where PD-L2 is predominant (1, 11).

**Figure 1:**
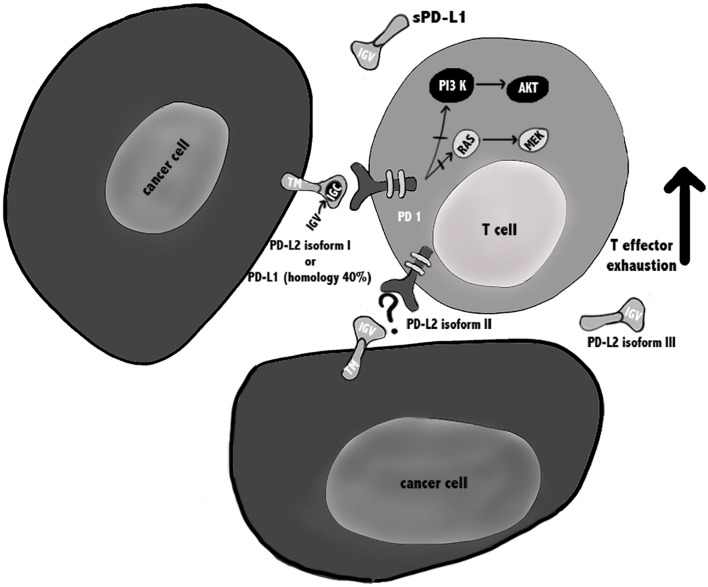
PD-1/PD-Ls pathways in cancer. PD-L1 is a type I transmembrane glycoprotein with a single N-terminal IgV-like domain and exists also in a soluble form sPD-L1 that retains the IgV-like domain. PD-L2 is a type I transmembrane protein containing an IgV-like domain and an IgC-like domain and three isoforms of PD-L2 have been described that might influence the outcome of the immune response. It is suggested that isoforms II and III should be able to interact with PD-1, but further confirmation is needed. During TCR cross-linking, PD-1 by interacting with its ligands, causes inhibition of PI3K/Akt/mTOR and Ras/MAPK/Erk pathways, leading to down-regulation of T cells metabolism, and exhausted T cells.

Regarding the PD-1/PD-L1 and PD-1/PD-L2 pathways involved in T cell immune evasion, different reports have been published, mainly regarding the biochemical signaling regulated by the PD-1/PD-L1. It was reported that the binding of PD-L1 to PD-1 may cause T cell apoptosis, anergy, exhaustion, and interleukin-10 (IL-10) expression, suggesting that PD-L1 can act as a defender for PD-L1^+^ cancer cells from CD8^+^ T cell–mediated lysis (12, 13) (Figure 1).

Regarding the PD-L2/PD-1 signaling pathways, it may not be biologically identical, since Repulsive Guidance Molecule B (RGMb) is also a binding partner for PD-L2 (14). Thus, the PD-L2 blockade may evocate different cellular responses, depending on the binding partner interaction, which can lead to potential varied biological outcomes. Up to now, in human anti-tumor immunity, the relationship between PD-1, PD-L1, and PD-L2 in their cellular expression profile and regulation, potential interactions and biological is considered not completely defined.

### PD-1 Ligands in the Tumor Microenvironment Influence the Anti-tumor Response

PD-L1 and PD-L2 are expressed in different cancer cells and in their microenvironment (4, 8), including infiltrating immune cells (15, 16). However, their prognostic value is still debated and the role they might play when expressed in the tumor microenvironment has not be fully elucidated yet (17).

Previous evidence shows that PD-L1 expression by cancer cells correlates with poor prognosis (18), while PD-L1 expression by tumor-infiltrating immune cells is associated with improved overall survival (OS) (16). Furthermore, it seems that PD-L1 expressed by APC, rather than cancer cells, is essential for the response to immune checkpoint blockade therapy (19). Specifically, survival analysis showed that the presence of PD-L1 on macrophages had a protective role and enhanced the prognosis of patients with hepatocellular carcinoma. Macrophages are involved in maintaining an active immune microenvironment, with high numbers of infiltrating CD8^+^ T cells and high immune-related gene expression levels (15).

Sepesi et al. investigated PD-L1 expression in surgically resected stage I non-small cell lung cancer and, in contrast, demonstrated that lower PD-L1 expression in the tumor, but also in tumor-infiltrating macrophages, was associated with significantly better OS (20).

The existence of conflicting reports about PD-L1 and−2 prognostic value can be generally attributed to technical disparities (e.g., variations in staining protocols across individual laboratories and use of different primary antibody clones to identify PD-Ls in tumor tissue), as well as different clinical features of the analyzed samples (site and size of cancer, treatments, follow-up time, etc.). Moreover, PD-L1 and−2 are dynamic markers that can be up- or downregulated over time, making their evaluation complicated (17, 21).

Direct activation of the PD-1 axis by cancer cells leads to a potent inhibitory signal in T lymphocytes resulting in anti-tumor immunity impairment and tumor cells ability to escape immunosurveillance (4, 19). Specifically, it has been shown that PD-1 activation inhibits glucose consumption, cytokine production, proliferation and survival in T lymphocytes, thus preventing the expression of transcription factors associated with effector T cell functions, such as GATA-3, T-bet, and Eomesodermin (Eomes) (4). PD-1/PD-Ls binding attenuates TCR-mediated signaling, thus impairing PI3K/Akt and Ras/MEK/Erk pathways, both required for T-cell activation (4).

PD-Ls are expressed in several solid tumors (8, 22), and immune checkpoint inhibitors, such as anti-PD-1 and anti-PD-L1 antibodies, showed efficacy in cancers with high mutational load, including lung cancer, melanoma, and microsatellite instable (MSI) tumors (23). It was shown that this efficacy is linked to the presence of tumor specific neoantigens that induce a Th1/CTL response that is counterbalanced by overexpression of multiple immune checkpoints such as PD-1/PD-L1 (23). In addition, PD-1/PD-L1 axis blockade might activate tumor-specific T lymphocytes to kill tumor cells by inducing TNF-α and IFN-γ (22).

For gynaecologic malignancies, the expression of PD-1 ligands has been reported in ovarian (17, 21, 22, 24–31), uterine (5–7, 32–38), cervical (23, 32, 39–50), and vulvar (32, 51–54) cancers, which we describe in detail in the next section.

## PD-1 and PD-Ls Expression in Endometrial Cancer

In normal endometrium the role of the immune system is extremely complex, since it must prevent sexually transmitted infections but should also be able to help the growth of an allogenic fetus during pregnancy (23). So far, few reports characterized PD-1 and its ligands' expression in gynecological cancer and data are quite controversial. The expression profile of these immune checkpoints has been analyzed predominantly by immunohistochemistry, in biopsies obtained from both healthy subjects and cancer patients.

### PD-1 in Endometrial Cancer

The PD-1 receptor has been found almost exclusively in immune cells infiltrating the tumor (32, 37, 38), and not in normal endometrium (5). Additionally, a deep analysis performed on 183 patients showed that high expression of PD-1 within and at the margins of a tumor, with a high PD-1/CD8^+^ ratio in the center, was associated with favorable OS (35).

Additional reports found a correlation between PD-1 expression in intraepithelial and peritumoural lymphocytes with DNA polymerase ε (POLE) mutation and MSI status of the patients (32, 37, 38). Specifically, it has been reported that PD-1 expression in tumor-infiltrating immune cells was more frequently found in moderately, poorly differentiated endometrial cancers, non-endometrioid type II (serous, clear cell, mucinous) endometrial cancers (5, 35, 36), and POLE and MSI subgroups (32, 37, 38).

### PD-L1 in Endometrial Cancer

Regarding PD-1 ligands, all data concordantly showed that PD-L1 is expressed in most of the analyzed specimens (5–7, 32–35, 37), predominantly located in the cytoplasm (5–7). Several studies showed that PD-L1 was expressed in a similarly high percentage of samples in both normal endometrium and endometrial tumors (5–7).

PD-L1 expression in cancer cells correlates with post-menopausal status, high histological grade (grade 3), deep myometrial invasion (≥1/2), lymphovascular invasion, adjuvant therapy, and MSI status (35). High PD-L1 immuno-reactivity on immune cells, and not on tumor cells, is an independent predictor of adverse progression-free survival (PFS) in all patients, including the microsatellite stable (MSS) subgroup (35). In addition, some reports evidenced that PD-L1 expression in intraepithelial immune cells was significantly more frequent in POLE mutant and MSI tumors, compared to MSS tumors (32, 37, 38), while PD-L1 expression in tumor cells did not differ between POLE mutant, MSI and MSS patients (32).

However, data regarding PD-L1 expression in cancer cells are controversial: one study showed that only 1 out of 116 tumors expressed PD-L1 on tumor cells, but this under-estimation could be linked with the use of tissue microarrays, since PD-L1 expression is known to be heterogenous (37).

Another study regarding gynecological samples, in 47 uterine sarcoma samples, found that PD-L1 expression was upregulated in comparison with normal endometrium, suggesting that this protein is a potential target for immunotherapy (7), while Bregar et al., using a smaller number of samples (10 patients), found that PD-L1 is expressed in only 30% of specimens (34).

### PD-L2 in Endometrial Cancer

For PD-L2 very few data are available so far, and its expression seems to differ from PD-L1, with no significant difference between normal endometrium and tumor (5–7).

High PD-L2 expression was shown in 30% of primary endometrial carcinoma patients and 16% of uterine sarcoma patients, demonstrating the potential of PD-L2 blockade in a limited proportion of uterine cancer patients (7). It has been shown that PD-L1 and PD-L2 expression was more frequent in moderately, poorly differentiated, non-endometrioid endometrial cancer and seems to be correlated with POLE and MSI status (5, 33, 36). Type II endometrial cancer and poorly differentiated histological features are generally associated with worse prognosis and, in addition, PD-1 axis expression suggests that it may cause immunosuppression to favor tumor growth, thus negatively affecting patients' survival (5).

## Expression of PD-1, PD-L1, and PD-L2 in Ovarian Cancer

Ovarian cancer is the most lethal disease among gynecological cancers (17, 22, 29–31) and is known to be an immunogenic tumor.

### PD-1 and PD-L1 in Ovarian Cancers

Some reports showed that PD-L1 expression is found in epithelial ovarian cancers (EOC) (17, 20, 21, 24–26, 30), especially in serous ovarian cancers (SOC) (28, 29), ovarian clear cell carcinomas (OCCC) and in malignant ascites (31), a sign of peritoneal carcinomatosis derived from ovarian cancer (22).

In a cohort of 122 patients with OCCC, Zhu et al. showed that 55 cases (44.7%), classified as having high PD-L1 expression (PD-L1^high^), were significantly associated with advanced stages (III–IV) (22). Cases with high PD-L1 and PD-1 expression showed significantly poorer PFS and OS, compared to those with low PD-L1/PD-1 expression (22, 24, 28, 29). In subgroup analysis, PD-L1^high^ was associated with poorer prognosis compared to PD-L1^low^ in platinum-resistant and advanced stages (III–IV) patients (22). Drake et al. analyzed 55 ovarian cancer biopsies and showed that PD-1 was detected in 87% of the tumors in both stroma and epithelium, while PD-L1 was only present in 33% of patients, exclusively in high-grade tumors (17). Additionally, they found that low density of PD-1 and PD-L1 expressing cells in tumor tissue was significantly associated with advanced disease, failing to show any significant association between survival and PD-1 or PD-L1 expression in ovarian cancer (17), while patients with recurrent tumors and increased infiltrating PD-1^+^ immune cells had longer OS (21). The correlation of PD-1 and PD-L1 expression with high-grade tumors and stage IV International Federation of Gynecology and Obstetrics (FIGO) disease has also been confirmed by other studies (28, 29).

Wieser et al. showed that, in a cohort of 158 patients with high-grade serous ovarian cancers, BRCA1/2 mutated tumors were characterized by high PD-1 expression, and that PD-L1 was observed mainly in BRCA1/2 and TP53 mutated cancers (29). Xiao et al. reported that PD-1 is expressed in tumor infiltrating lymphocytes and PD-L1 in tumor cells and in intratumoural immune cells, but there was no significant difference of PD-1^+^ intratumoural immune cells in tumors with different mismatch repair (MMR) status (30). MSI ovarian cancers exhibited a significantly higher number of PD-L1^+^ intratumoural immune cells compared to MSS ovarian cancers, while PD-L1 expression was not different in tumors, irrespectively from their MMR status (30).

In addition, no significant difference regarding PD-L1 expression in tumor cells and tumor infiltrating lymphocytes, and PD-1 expression in infiltrating lymphocytes, has been found between primary and recurrent disease (21).

### PD-L2 in Ovarian Cancers

So far, only few studies investigated the expression of PD-L2 in ovarian cancer. An analysis on 70 patients showed that PD-L2 expression was not related to patient prognosis or other clinical variables, but negatively correlated with the number of FOXP3^+^ T regulatory cells (Tregs) (24). Imai et al. analyzed the expression of PD-L1 and PD-L2 on tumor cells and APCs in malignant ascites from epithelial ovarian cancer patients (31), and found differential PD-L1 expression in tumor cells between patients with high or low PD-1-expressing CD4^+^ T cells (43.9 and 27.3%, respectively), while no difference in PD-L1 expression was observed between patients with high and low PD-1 expression on CD8^+^ T cells (34.1 and 27.3%, respectively). Between 2.3 and 3.2% of the patients with high or low PD-1 on CD4^+^ T cells and CD8^+^ T cells also expressed PD-L2. No correlation was found between PD-L1/2 expression and clinical variables or outcomes (31).

To support a potential role of PD-1 and PD-L1/ PD-L2 axis as targets in ovarian cancer, it has been reported in syngeneic orthotopic mouse model of epithelial ovarian cancer, that treatment with anti-PD-1 or anti-PD-L1 antibodies resulted in tumor rejection in 75% of the treated-mice, while mice treated with anti-PD-L2 antibody did not reject tumors (25). These data can be explained considering the selected models that expressed lower levels of PD-L2 than PD-1 and PD-L1. Additionally, PD-1 and PD-L1 blockade significantly increased the CD8^+^ to Tregs and CD4^+^ to Tregs ratios within the tumor, while, on the contrary, there was no significant change in the CD8^+^ or CD4^+^ to Tregs ratios (25).

## Expression of PD-1, PD-L1, and PD-L2 in Other GYNECOLOGICAL Cancers

Cervical cancer is the third most common gynecological malignancy in Europe (23). Little information is available, up to now, regarding the expression of PD-1 ligands (23, 32, 39, 43–47).

A report from Howitt et al. showed that cervical cancer is a potential candidate for clinical trials testing PD-1 blockade (23, 32, 39). In fact, using FISH analysis on 48 Formalin-Fixed Paraffin-Embedded (FFPE) tissue specimens of cervical squamous cell carcinoma, they observed co-amplification or co-gain of PD-L1 and PD-L2 in 32 out of 48 cases (67%). Immunohistochemical staining for PD-L1 revealed high expression in 95% of the tumors with membranous staining pattern (32).

Persistent infection with human papilloma virus (HPV) is an essential step in the development of most cervical cancers (40). Some studies hypothesized that HPV may activate PD-1/PD-L1 to evade host immune responses, resulting in persistence of the cervical intraepithelial neoplasia (41). The identification of HPV as an etiological factor leads to antigen production and presentation, thereby making cervical cancer immunogenic (42). Recently, the role of the PD-1/PD-L1 axis in HPV associated head and neck squamous cell cancer (HPV-HNSCC) creating an “immune-privileged” site for initial viral infection and subsequent adaptive immune resistance suggests a rationale for therapeutic blockade of this pathway in patients with HPV-associated tumors (43). Significant PD-L1 expression in cervical carcinoma has been confirmed in several studies (44–47). As a consequence, this immunogenic disease requires a highly immunosuppressive microenvironment to progress and metastasize (48, 49) which has been demonstrated in tumor-positive lymph nodes where high Treg levels, low CD8^+^ T cell/Treg ratio and high levels of PD-L1^+^ and HLA-DR^+^ myeloid cells were found (50).

Regarding another gynecological malignancy, vulvar cancer, the clinical relevance of PD-L1 expression has not been completely studied so far (32).

Although rare, incidence rates of vulvar cancer are increasing and, in locally advanced, metastatic or recurrent disease, prognosis is poor and new treatment modalities are needed (51). Screening of 23 vulvar squamous cell carcinomas revealed 6 cases (26%) with co-amplification of PD-1 ligands, 4 cases (17%) showed co-gain, 6 cases (26%) showed polysomy, and 7 cases (30%) showed disomy. Immunohistochemical staining for PD-L1 across all cases revealed the highest median PD-L1 protein expression in cases with co-amplification of PD-L1 and PD-L2, and decreasing values with decreasing genetic complexity (32). Previous studies showed that PD-L1 is expressed in the majority of vulvar squamous cell carcinoma samples (51–54), in both cancer cells and peritumoural immune cells (52–54). Additionally, its expression was related with several components of immune system (CD3^+^, CD20^+^, and CD68^+^ intra-tumor immunocytes) (51, 54), while a significant correlation with immunosuppressive cell populations (FOxP3^+^ Treg cells) was reported only by Sznurkowski et al. (54). Data analyzing the clinical impact of PD-L1 expression in vulvar cancer reveal that it is not clear whether its expression correlates with clinicopathological parameters.

In summary, no significant associations were observed between PD-L1 presence and typical clinicopathological factors (51), except for tumor stage as reported by Sznurkowski et al. (54), and PD-L1 expression occurs more often in high risk HPV-negative samples (51). Regarding survival analysis, it is reported that PD-L1 expression did not influence the OS (51, 53), but patients with primary tumors positive for immune cells-PD-L1 expression had improved OS compared to negative ones (54).

The presence of PD-L1 also seems to be an independent prognostic factor for recurrence free survival (51).

## Ongoing Immunotherapy Clinical Trials in GYNECOLOGICAL Malignancies

Several clinical trials are ongoing at the moment, according to the ClinicalTrials.gov database [accessed July 06, 2019], testing anti-PD-1/PD-L1 blockade alone or in combination in patients with endometrial, cervical, vulvar and ovarian cancer, while there are no ongoing clinical trials using anti-PD-L2 (Tables 1–3).

**Table 1:** Ongoing immunotherapy clinical trials for patients with endometrial cancer.

**ClinicalTrials.gov identifier**	**Status**	**Interventions/alone or in combination**	**Phase**
NCT02630823	Active, not recruiting	Pembrolizumab (anti-PD-1) + Paclitaxel/Carboplatin/Radiation (standard of care)	I
NCT02725489	Active, not recruiting	Durvalumab (anti-PD-L1)	II
NCT02728830	Active, not recruiting	Pembrolizumab (anti-PD-1)	Early I
NCT02646748	Active, not recruiting	Pembrolizumab (anti-PD-1) + itacitinib/INCB050465	I
NCT02914470	Active, not recruiting	Atezolizumab (anti-PD-L1) + cyclophosphamide/Carboplatin	I
NCT02521844	Active, not recruiting	Pembrolizumab (anti-PD-1) + ETC-1922159	I

**Table 2:** Ongoing immunotherapy clinical trials for patients with ovarian cancer.

**ClinicalTrials.gov identifier**	**Status**	**Interventions (alone or in combination)**	**Phase**
NCT02608684	Active, not recruiting	Pembrolizumab (anti-PD-1) + Gemcitabine/Cisplatin	II
NCT02728830	Active, not recruiting	Pembrolizumab (anti-PD-1)	Early I
NCT03287674	Active, not recruiting	Nivolumab (anti-PD-1) + Cyclophosphamide/Fludarabine/TIL infusion/Interleukin-2/Ipilimumab	I/II
NCT03277352	Active, not recruiting	Pembrolizumab (anti-PD-1) + INCAGN01876/Epacadostat	I/II
NCT03312114	Active, not recruiting	Avelumab (anti-PD-L1)	II
NCT02674061	Active, not recruiting	Pembrolizumab (anti-PD-1)	II
NCT03029598	Active, not recruiting	Pembrolizumab (anti-PD-1) + Carboplatin	I/II
NCT02335918	Completed	Nivolumab (anti-PD-1) + varlilumab	I/II
NCT02915523	Active, not recruiting	Avelumab (anti-PD-L1) + entinostat	I/II
NCT02452424	Completed	Pembrolizumab (anti-PD-1) + PLX3397	I/II
NCT02644369	Active, not recruiting	Pembrolizumab (anti-PD-1)	II
NCT03073525	Active, not recruiting	Atezolizumab (anti-PD-L1)	II
NCT02526017	Active, not recruiting	Nivolumab (anti-PD-1) + FPA008	I
NCT02580058	Active, not recruiting	Avelumab (anti-PD-L1) + PLD	III
NCT03365791	Active, not recruiting	PDR001 (anti-PD-1) + LAG525	I
NCT02764333	Active, not recruiting	Durvalumab (anti-PD-L1) + TPIV200	II
NCT02431559	Active, not recruiting	Durvalumab (anti-PD-L1) + Pegylated Liposomal Doxorubicin	I/II
NCT02914470	Active, not recruiting	Atezolizumab (anti-PD-L1) + carboplatin, cyclophosphamide	I
NCT02725489	Active, not recruiting	Durvalumab (anti-PD-L1)	II
NCT01975831	Active, not recruiting	MEDI4736 (anti-PD-L1) + Tremelimumab	I
NCT03038100	Active, not recruiting	Atezolizumab (anti-PD-L1) + Carboplatin/Atezolizumab/Bevacizumab	III
NCT01772004	Active, not recruiting	Avelumab (anti-PD-L1)	I/II
NCT03574779	Active, not recruiting	TSR-042 (anti-PD-1) + Niraparib/Bevacizumab	II
NCT02521844	Active, not recruiting	Pembrolizumab (anti-PD-1) + ETC-1922159	I

**Table 3:** Ongoing immunotherapy clinical trials for patients with cervical cancer.

**ClinicalTrials.gov Identifier**	**Status**	**Interventions**	**phase**
NCT01975831	Active, not recruiting	MEDI4736 (anti-PD-L1) + Tremelimumab	I
NCT02914470	Active, not recruiting	Atezolizumab (anti-PD-L1) + Carboplatin/Cyclophosphamide	I
NCT02725489	Active, not recruiting	Durvalumab (anti-PD-L1)	II
NCT02921269	Active, not recruiting	Atezolizumab (anti-PD-L1) + Bevacizumab	II
NCT02257528	Active, not recruiting	Nivolumab (anti-PD-1)	II
NCT03073525	Active, not recruiting	Atezolizumab (anti-PD-L1)	II

Clinical trials data were collected from ClinicalTrials.gov database, selecting only completed trials or in “Active, not recruiting” status.

### Endometrial Cancer

Regarding endometrial cancer, 6 clinical trials are ongoing (Table 1). Most of them are Phase I clinical trials and preliminary results, reported by the American Society of Clinical Oncology (asco.org), showed that atezolizumab (anti-PD-L1), and pembrolizumab (anti-PD-1) might be promising agents for endometrial cancer treatment.

Most relevant results showed that in a phase I study, 15 patients eligible based on PD-L1 status (>5% of positivity in tumor-infiltrating immune cells) were treated with atezolizumab and evaluated for safety and efficacy. Results showed that atezolizumab had a favorable safety profile and 13% (2/15) of patients showed a reduction in tumor size. A trend for higher PFS and OS has been observed in patients with high levels of tumor-infiltrating immune cells. Clinical benefit appeared to increase with higher PD-L1 expression, suggesting a link between PD-L1 status and response to atezolizumab. In addition, hypermutation, and/or high immune infiltration may be linked to response to PD-L1 blockade (Clinical trial information: NCT01375842) (55).

In a different phase I clinical trial, pembrolizumab was administered in 24 patients with endometrial carcinoma (excluding sarcomas), failure of prior systemic therapy, and PD-L1 expression in ≥1% of tumor or stromal cells. A reduction in tumor size was confirmed in 13.0% of the patients, while 3 patients achieved stable disease. PFS and OS rates were 19.0 and 68.8%, respectively. In conclusion, Pembrolizumab demonstrated an acceptable safety profile and anti-tumor activity (Clinical trial information: NCT02054806) (56).

### Ovarian Cancer

For ovarian cancer 22 clinical trials are ongoing, 2 of which are completed (Table 2). Some of the early-phase clinical trials of anti-PD-1 or anti-PD-L1 antibodies have shown good safety profiles and durable anti-tumor response in certain patient population(s). However, their response rates remain between 10 and 15% (31, 57). Available interim reports from some of the trials show promising objective response rates (ORR) for the treatment of ovarian cancer with nivolumab (anti-PD-1) (ORR of 15%, *n* = 20 patients), pembrolizumab (ORR 11.5%, *n* = 49), or avelumab (anti-PD-L1) (ORR 10%, *n* = 124) (17, 58, 59). Preliminary data presented at the annual ASCO meeting in 2016 of a phase I trial evaluating durvalumab (anti-PD-L1) in combination with olaparib (PARP inhibitor), showed a disease control rate (DCR) of 67% for the doublet olaparib - durvalumab in a cohort including BRCA wild type triple negative breast cancer and EOC cases (23).

In the KEYNOTE-28 trial, which explored the activity of pembrolizumab in several solid tumors, outcome of ovarian cancer was ORR of 11.5%, and only 23.1% showed tumor shrinkage from baseline (57).

### Cervical Cancer

For cervical cancer, 6 clinical trials are ongoing (Table 3). Most relevant findings showed that in a phase Ib study with 24 patients affected by advanced cervical squamous cell cancer and PD-L1 expression in ≥1% of tumor or stromal cells, pembrolizumab was well-tolerated and showed promising anti-tumor activity (Clinical trial information: NCT02054806) (60), while its clinical benefit was investigated in the phase 2 KEYNOTE-158 trial. Pembrolizumab administration has been also investigated in a single cohort trial enrolling 98 patients with recurrent or metastatic cervical cancer, expressing PD-L1 with a positive ratio of the number of all PD-L1–expressing cells (tumor cells, lymphocytes, macrophages) to the number of all tumor cells, or a Combined Positive Score (CPS) ≥1. The ORR in 77 patients was 14.3% (95% CI: 7.4, 24.1), including 2.6% complete responses and 11.7% partial responses. No responses were observed in patients with tumors negative for PD-L1 expression (CPS <1). Serious adverse reactions occurred in 39% of patients (Clinical trial information: NCT02628067) (61).

On June 12th 2018, pembrolizumab was approved by Food and Drug Administration (FDA), for treatment of patients with recurrent or metastatic cervical cancer, expressing PD-L1 (CPS ≥1) as determined by an FDA-approved test, with disease progression on or after chemotherapy^1^.

**Figure 2:**
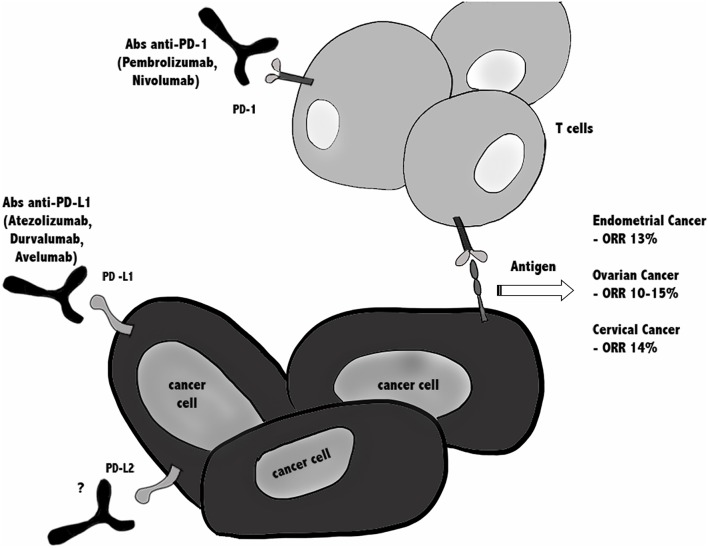
Immunotherapy against PD-1/PD-Ls in gynecological cancers. Blocking the PD-1/PD-L1 immune checkpoint pathway by anti-PD-1 or anti-PD-L1 antibodies suppresses cancer cell survival and enhances the antitumor responses of T cells, leading to tumor regression and rejection. Actually, several clinical trials are ongoing testing anti-PD-1/PD-L1 blockade alone or in combination, in patients with endometrial, cervical, vulvar, and ovarian cancer, while there are no ongoing clinical trials using anti- PD-L2. In all gynecological cancers ORR is around 10–15%, argues for combinatorial treatments are taken in consideration.

In conclusion, since in all gynecological cancers ORR is around 10–15%, this emphasizes the need for combination treatments to improve efficacy of immune checkpoint (Figure 2).

## Future Directions for Immune Checkpoint Inhibitors (ICIs) Combination Therapies

Albeit ICIs therapies have been shown to induce durable responses and long-term remission in several cancer types, many patients fail to respond, develop resistance over the time or show immune-related adverse effects (62–65). The unresponsiveness or the toxicity of ICIs represents a strong rationale for the combination of ICIs with other treatments to increase the response rate of non-immunological tumors. For example, therapeutic approaches that induce the release and presentation of tumor antigens could be able to foster a *de novo* anti-tumor T cell response. In this regard, candidates for a combination therapy with ICIs could be cancer vaccines, oncolytic viruses, radiation, or low-dose chemotherapy (66).

Another potential combination approach with ICIs could be with bispecific antibodies, which recruit patient's T cells or NK cells against cancer cells expressing tumor-associated antigens. An example came from hematologic malignancies, wherein a bispecific antibody targeting both CD3 and CD123 (67, 68) was used but showed benefit in only a small fraction of patients. A major mechanism limiting the therapeutic efficacy was T cell anergy and exhaustion driven by ICIs pathways (mainly PD-L1/PD-1) (69). Inspired by this inhibitory role of ICIs pathway, combining ICIs with bispecific antibodies showed enhanced T cell proliferation and IFN-γ production (70).

One more possibility to improve ICI efficacy might be combination with cytokine therapy. The cytokine IL-2 has been approved for the treatment of metastatic renal cell carcinoma and advanced melanoma but is accompanied by severe side effects (71). However, modified IL-2 formulations such as bempegaldesleukin (NKTR-214) have an improved safety profile and have shown capabilities of enhancing the proliferation and activation of CD8^+^ T cells and NK cells without increasing the number of Tregs (72). Recently, the PIVOT-02 trial (combination of NKTR-214 and nivolumab) has shown that this combination is safe and efficacious (ORR 48% in 23 patients) in metastatic urothelial carcinoma (73).

In addition, a recent study has demonstrated that DC-derived IL-12 is necessary for successful anti-PD-1 cancer therapy, suggesting that IL-12 and ICIs could be rationally combined (74).

Finally, there is strong rationale to combine anti-angiogenic therapies with ICI's, since anti-angiogenic therapies induce a normalization of the tumor vasculature, which leads to enhanced infiltration of T lymphocytes in the tumor.

## Conclusion

Cancer immunotherapy is emerging as a promising component for cancer therapy. The most promising immunotherapy that showed good results involves antibodies targeting inhibitory immune checkpoint molecules (75).

Results obtained for patients with non-small cell lung cancer, renal cancer, and melanoma are evident and encouraging. However, in gynecological malignancies many aspects remain controversial in preclinical and clinical studies (23). Uncertain is the selection of patients because objective response rates remain low and retrospective analysis on biopsies showed opposing results for OS and PFS in patients with similar pattern of expression of PD-1 and its ligands (15, 17, 20–22, 24, 28, 29, 32, 34).

Regarding the second ligand PD-L2, it is needed to better clarify its role inside tumor microenvironment, together with the evaluation of other biological markers, in order to improve the efficiency of immunotherapy malignancies of the female genital tract.

## Author Contributions

OM, DA, CA, MN, FA, and ST wrote the paper. MM, GS, CA, and FM have revised the clinical trials and the paper.

### Conflict of Interest

The authors declare that the research was conducted in the absence of any commercial or financial relationships that could be construed as a potential conflict of interest.

## Footnotes

^1^Merck & Co. Press Release Details. https://investors.merck.com/news/press-release-details/2018/FDA-Approves-Mercks-KEYTRUDA-pembrolizumab-for-Previously-Treated-Patients-with-Recurrent-or-Metastatic-Cervical-Cancer-Whose-Tumors-Express-PD-L1-CPS-Greater-Than-or-Equal-to-1/default.aspx
